# Macromolecular crystallography and biology at the Linac Coherent Light Source

**DOI:** 10.1107/S1600577525002735

**Published:** 2025-04-23

**Authors:** Sandra Mous, Mark S. Hunter, Frédéric Poitevin, Sébastien Boutet, Leland B. Gee

**Affiliations:** a Linac Coherent Light Source SLAC National Accelerator Laboratory Menlo Park CA94025 USA; University of Manchester, United Kingdom

**Keywords:** X-ray free-electron lasers, serial femtosecond crystallography, protein crystallography, time-resolved crystallography, room-temperature crystallography

## Abstract

The Linac Coherent Light Source (LCLS) has significantly impacted the field of biology by providing advanced capabilities for probing the structure and dynamics of biological molecules with high precision. The ultrashort coherent X-ray pulses from the LCLS have enabled ultrafast, time-resolved, serial femtosecond crystallography that is inaccessible at conventional synchrotron light sources. Since the facility’s founding, scientists have captured detailed insights into biological processes at atomic resolution and fundamental timescales. The ability to observe these processes in real time and under conditions closely resembling their natural state is transforming our approach to studying biochemical mechanisms and developing new medical and energy applications. This work recounts some of the history of the LCLS, advances in biological research enabled by the LCLS, key biological areas that have been impacted and how the LCLS has helped to unravel complex biological phenomena in these fields.

## Abstract 2

The significant advancements in biological research enabled by the Linac Coherent Light Source (LCLS) are explored. By offering ultra-bright, coherent X-ray pulses with femtosecond durations, LCLS has revolutionized the study of biological molecules and their dynamic processes. Techniques such as serial femtosecond crystallography leverage these unique properties, allowing researchers to image complex biomolecular structures at atomic resolutions and in near-native conditions. The ability to perform time-resolved studies and observe real-time interactions at the molecular level provides deeper insights into mechanisms underlying biological functions. The discussions presented highlight key studies and innovations facilitated by LCLS, underlining its critical role in advancing both fundamental biological knowledge and applied sciences such as drug development.

## 1. Introduction

Advancements in coherent X-ray light sources have created unique opportunities for the biological sciences. The construction of the Linac Coherent Light Source (LCLS) and subsequent development of serial femtosecond crystallography (SFX) have enabled researchers to obtain significant insights into biological structures and processes at unprecedented resolutions and timescales. These breakthroughs are made possible by X-ray free-electron lasers (XFELs) like the LCLS, which provide brilliant pulses of temporally short X-rays with remarkable coherence and brightness. This article explores the transformative impact of LCLS on biological research, highlighting key studies and current/future innovations that underscore its contributions to the field and potential for the future.

## 2. The promise of free-electron lasers in biology

The use of synchrotron radiation for structural biology has been extremely successful for decades since its first demonstration by Hodgson and coworkers (Phillips *et al.*, 1976 ▸). Methods have been developed, optimized and perfected to achieve great efficiency in rapidly solving structures of biomolecules. Through the years, new X-ray sources were developed with ever increasing brightness to allow faster measurements (Helliwell, 2011 ▸). Unfortunately, while brighter beams can decrease measurement times, there is a fundamental limit to how many X-rays can be used to probe a sample in a continuous measurement before the very same X-rays significantly damage the structures being measured. For hard X-rays with wavelengths typically used for structural biology, the absorption cross-section is roughly an order of magnitude larger than the scattering cross-section and therefore there are more X-rays that damage the sample than there are X-rays that provide useful information.

It was recognized in 1999 (Neutze *et al.*, 2000 ▸) that it is possible to overcome this damage limit in structural biology when measurements can be made temporally shorter than pulse widths offered at synchrotrons of the time. Thus, if a single X-ray pulse can be made short enough to interact with the sample before damage can occur, it is possible to increase the intensity of that pulse probing the sample and increase the measured signal. This allows, for example, smaller objects to be measured, *i.e.* smaller crystals or more weakly diffracting crystals than what is conventional at synchrotrons. It can also allow molecules that are particularly sensitive to radiation damage, such as metalloenzymes, to be measured more accurately. The concept of probing before destruction, enabling crystallography with smaller crystals, room-temperature measurements and possibly measuring down to single biomolecules, promised unique new opportunities for novel XFEL sources that can produce intense pulses of X-rays a few tens of femtoseconds in duration, or even shorter.

## 3. The birth of LCLS

Although the theory of short-pulsed XFEL radiation was well established by the late 20th century, the practical feasibility, especially for generating pulses with wavelengths below ∼1 Å, was not readily attainable. Early theoretical studies in the 1980s identified that contemporary storage rings were inadequate to support the low electron beam emittance required to achieve wavelengths shorter than the XUV regime (Murphy & Pellegrini, 1985 ▸).

In the early 1990s, work on the Stanford linear collider (SLC) at SLAC National Accelerator Laboratory revealed the feasibility of compressing electron beams without compromising beam emittance. This progress culminated in a Stanford Synchrotron Radiation Lightsource (SSRL) workshop at SLAC in February 1992, where utilizing the SLAC linear accelerator to produce a sub-nanometre SASE (self-amplified spontaneous emission) XFEL was proposed (Pellegrini, 1992 ▸).

From 1992 to 1995, a specialized working group identified key technological developments essential for achieving such a short-wavelength XFEL (Winick *et al.*, 1994 ▸). Keith Hodgson was a member of the initial 19-member study group that outlined the scientific justifications for such a light source, first named the Linac Coherent Lightsource (LCLS) in a memorandum dated 13 June 1992 (Pellegrini, 2012 ▸).

Realization of a hard XFEL faced significant challenges in 1994, as the project’s scale and costs were considered insurmountable by the National Research Council, necessitating further research and development to reduce expenses (Levy *et al.*, 1994 ▸). Furthermore, there was concern that the anticipated powerful X-ray pulses might destroy samples before useful information could be obtained (Pellegrini, 2012 ▸). By the end of 1997, sufficient progress had been made for the United States Department of Energy (DOE) to consider funding further research and development efforts towards creating a dedicated XFEL facility. In 1999, a DOE panel hosted a workshop with multiple sessions presenting the newly accessible technical developments and scientific potential for a <1 Å XFEL. Hodgson led a concluding discussion session, advocating for the LCLS, and the panel subsequently recommended funding the LCLS project (Leone *et al.*, 1999 ▸). A conceptual design was developed and finalized by 2002.

The project was then formalized with an engineering project management structure initially overseen by Hodgson in his role as director of SSRL. Construction of the accelerator apparatus and undulators was completed by 2009, with key performance experiments demonstrating XFEL lasing down to 1.5 Å in April of that year (Emma, 2009 ▸).

## 4. The early years of LCLS

**Figure 1:**
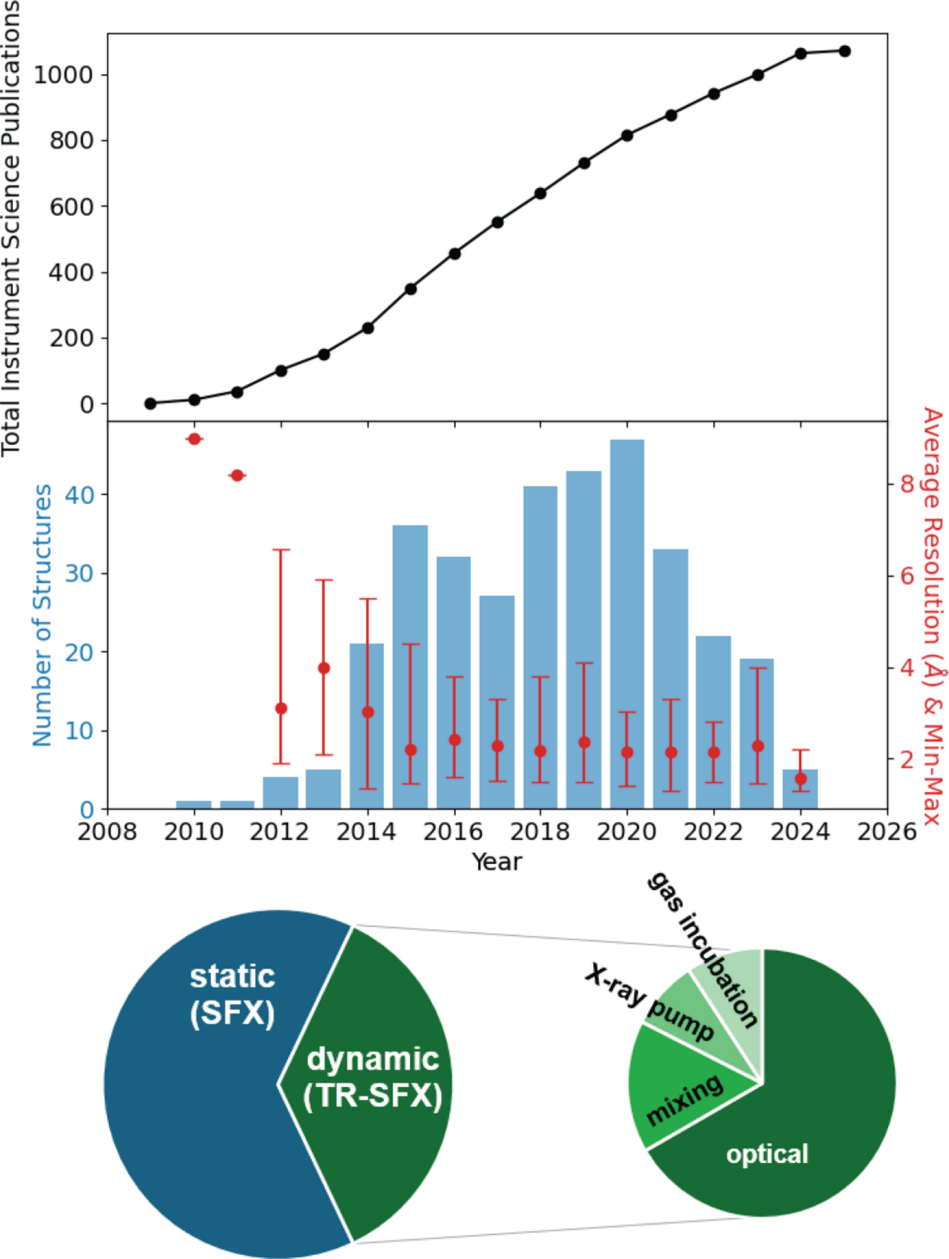
Top: cumulative publications from science at the LCLS scientific instruments including soft X-ray science. Middle: structures deposited in the Protein Data Bank (PDB) based on LCLS research. Bottom: contribution of static (SFX) and dynamic (TR-SFX) structures to the total number of depositions in the PDB. For dynamic structures, this number includes multiple conformations of a single protein, which could lead to an increased relative fraction of dynamic structures compared with static structures in the chart. A significant majority of TR structures rely on an optical laser pulse for triggering. Note that dynamic studies using the X-ray pump X-ray probe technique mainly focused on studying radiation damage-induced dynamics.

Following and concomitant to the successful demonstration of XFEL lasing at the LCLS, multiple specialized scientific instruments were developed at LCLS under the auspices of the LCLS Ultrafast Scientific Instruments (LUSI) project and related programs. For hard X-rays, the X-ray Pump–Probe (XPP) (Chollet *et al.*, 2015 ▸), X-ray Correlation Spectroscopy (XCS) (Alonso-Mori *et al.*, 2015 ▸), Coherent X-ray Imaging (CXI) (Boutet & Williams, 2010 ▸; Liang *et al.*, 2015 ▸) and Matter in Extreme Conditions (MEC) (Nagler *et al.*, 2015 ▸) instruments were developed, with XPP being the first instrument to receive a hard XFEL beam for user operations in 2010. Implied in their naming scheme, each of the instruments specialized in unique scientific disciplines, although this demarcation was non-exclusive as some science could be executed at more than one instrument for practical and/or logistical reasons. Leveraging this flexibility, by 2014 biological crystallography was considered ‘the hands-down winner of the early LCLS science race...’ by former LCLS director Joachim Stöhr (Roberts, 2014 ▸). Within five years the LCLS generated around 240 publications (Fig. 1 ▸).

Biological SFX research was initially concentrated at CXI before being complemented by the macromolecular femto­second crystallography (MFX) (Sierra *et al.*, 2019 ▸) instrument in 2016 (*vide infra*). MFX was conceptualized with contributions from the Structural Molecular Biology (SMB) group at SSRL, serving as a notable example of the synergies enabled by the colocation of the two light sources.

## 5. LCLS impact on biological sciences

The following sections discuss the historical contexts and where LCLS has had a transformative impact on in three key areas of structural biology: membrane proteins, metalloproteins and structural dynamics. These sections highlight how unprecedented insights into conventionally challenging membrane protein structures have been achieved, how metalloproteins and their intricate roles in various biological mechanisms have been elucidated in a damage-free manner, and how fast and ultrafast structural dynamics of biomolecules have been observed in real time. The detailed chemical and biological insights from these areas underscore the advancements enabled by LCLS, highlighting its critical role in advancing both fundamental biological knowledge and applied science fields such as synthetic biology and green chemistry.

### 5.1. Membrane proteins

X-ray crystallography has provided exciting insights into the structure and function of membrane proteins over the years. After the first membrane protein crystal structure was determined in 1985 (Deisenhofer *et al.*, 1985 ▸), the field has made significant advances. One substantial breakthrough was the development of lipidic cubic phase (LCP) crystallography (Landau & Rosenbusch, 1996 ▸). It has been suggested that the LCP medium mimics the native lipid bilayer, stabilizing the membrane proteins and allowing for an increased number of protein–protein contacts in the crystal structure. As a result, membrane protein crystals grown in an LCP matrix are more highly ordered than crystals obtained from solubilized membrane proteins, allowing true atomic resolution diffraction data to be collected (*e.g.* Borshchevskiy *et al.*, 2022 ▸). However, crystal growth in the LCP is typically constrained to two dimensions and limited by the diffusion speed of the proteins in the lipid bilayer due to the structure of this viscous medium (Caffrey, 2015 ▸). As a result, membrane proteins in the LCP matrix tend to grow 2D crystals that are only a few micrometres in size. Because of the small crystal size, determining the structure of membrane proteins using X-ray crystallography remains highly challenging, as the limited size aggravates the effects of radiation damage on the data quality while reducing Bragg peak intensities.

**Figure 2:**
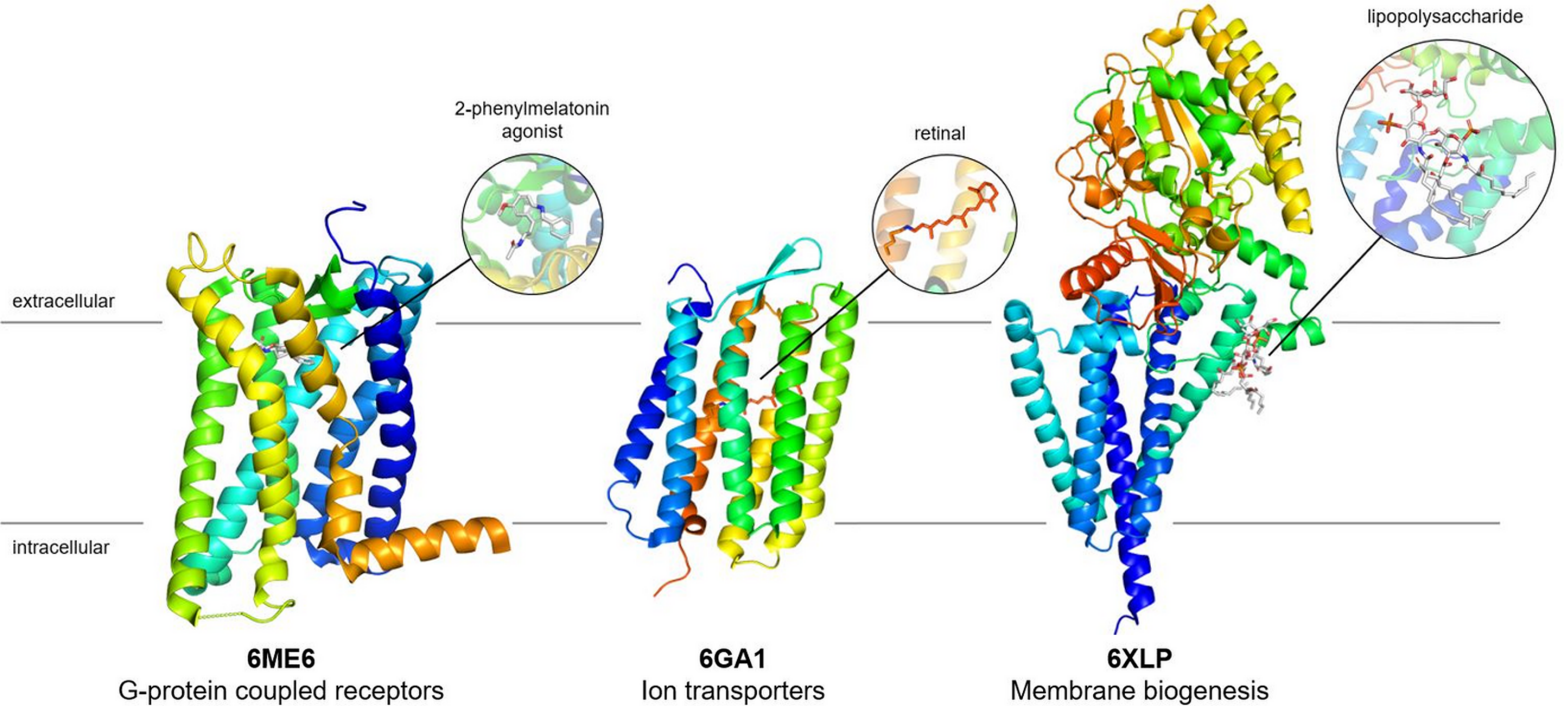
Highlights of various membrane protein structures obtained at the LCLS include G-protein coupled receptors (Johansson *et al.*, 2019 ▸), retinal-bound ion transporters (Nass Kovacs *et al.*, 2019 ▸) and membrane-bound proteins essential for the regulation of lipopolysaccharide biogenesis, with implications for antimicrobial drug design. PDB access codes are displayed underneath the structures.

X-ray crystallography at XFELs has been a great success in the field of membrane protein structural biology, as the extremely high brightness of XFEL radiation has enabled the structures of these proteins to be solved from small protein crystals (Fig. 2 ▸). While the intense X-ray pulses increase the structure factor amplitudes, enabling a diffraction signal to be captured from even nanometre-sized crystals (Chapman *et al.*, 2011 ▸), the ionizing radiation also destroys the crystal shortly following the exposure (Neutze *et al.*, 2000 ▸). Therefore, a new crystal needs to be delivered to the X-ray interaction point before the subsequent XFEL pulse arrives. This technique, known as SFX, effectively mitigates the accumulation of radiation damage as each crystal is exposed to the X-ray beam only once. Coupled with the ultrashort pulse duration, which enables the diffraction data to be captured before radiation damage can propagate throughout the crystal, the serial crystallography approach is especially suitable to samples that are sensitive to ionizing radiation, such as small-membrane protein crystals. Moreover, through avoiding the accumulation of radiation damage, structures can be determined at room temperature, revealing conformational flexibility critical for ligand binding and catalysis (Fraser *et al.*, 2011 ▸).

The first membrane protein structure determined with the SFX method was reported in 2013 and provided novel insights into the flexibility of the human serotonin 5-HT_2B_ receptor (Liu *et al.*, 2013 ▸). The method has since greatly contributed to our understanding of the structure and function of a diverse group of G-protein coupled receptors at near-physiological temperatures. Notable examples include medically relevant structures of protein–ligand complexes of angiotensin receptors (Zhang *et al.*, 2015 ▸; Zhang *et al.*, 2017*a* ▸); the human glucagon receptor (Zhang *et al.*, 2017*b* ▸); the MT1 and MT2 melatonin receptors (Stauch *et al.*, 2019 ▸; Johansson *et al.*, 2019 ▸); and PbgA, an inner membrane protein that plays a key role in lipopolysaccharide biosynthesis and *Salmonella* pathogenesis (Clairfeuille *et al.*, 2020 ▸). The rhodopsin photoreceptors, transporters and channels are another successful example of a class of membrane proteins studied with SFX, and studies have reported the structure and dynamics of a wide range of eukaryotic (Kang *et al.*, 2015 ▸; Oda *et al.*, 2021 ▸; Gruhl *et al.*, 2023 ▸), archaeal (Nango *et al.*, 2016 ▸; Nogly *et al.*, 2018 ▸; Nass Kovacs *et al.*, 2019 ▸) and bacterial (Skopintsev *et al.*, 2020 ▸; Yun *et al.*, 2021 ▸; Mous *et al.*, 2022 ▸; Hosaka *et al.*, 2022 ▸) homologues.

Following the success of SFX, room-temperature crystallography at synchrotron light sources has seen a revival in recent years. Serial synchrotron crystallography (SSX) has benefited from SFX technique development (Weinert *et al.*, 2017 ▸), while being further accelerated by the continuing development of the synchrotron light sources [most notably, the upgrade to diffraction limited storage rings (Eriksson *et al.*, 2014 ▸)], high repetition rate detectors (Tolstikova *et al.*, 2019 ▸) and new sample delivery methods (Zhao *et al.*, 2019 ▸). In light of these developments, it is anticipated that room-temperature membrane protein structure determination will become increasingly routine at synchrotron light sources. This will enable XFELs such as LCLS to focus their continued development on determining the structure of extremely radiation-sensitive proteins, such as the metalloenzymes described below, and capturing the ultrafast chemical dynamics in, for example, photosensitive proteins.

### 5.2. Metalloproteins

Nature facilitates many chemical transformations and biochemical processes through enzymes, proteins and their active site structure–activity relationships. However, certain activities can only be enabled by the unique electronic and geometric structures accessible to transition metals. These metals can exist as single metal centers, such as the Fe-containing electron-transfer protein Rubredoxin (Phillips *et al.*, 1977 ▸; Sun *et al.*, 2010 ▸) or the analogous Cu protein Azurin (Tullius *et al.*, 1978 ▸). These single metals can also be enclosed by macrocycles, which facilitate finer tuning of the metal’s electronic structure (Hocking *et al.*, 2007 ▸). This tuning enables activities such as oxygen transport in globins (Yan *et al.*, 2019 ▸; Wilson *et al.*, 2013 ▸), metabolism of xenobiotics through cytochrome P450 enzymes (Dey *et al.*, 2009 ▸) via an iron heme-based cofactor, methane conversion in archaea through a nickel corrin metallocofactor (Sarangi *et al.*, 2009 ▸) and various metabolic transformations facilitated by the cobalt-containing cobalamin vitamin (Banerjee & Ragsdale, 2003 ▸).

Moreover, metallocofactors can become significantly more diverse in the case of dimetallic cofactors and complex mixed metal clusters. The intricate electronic and geometric structures of these systems enable much more unique functionality. For example, the heteronuclear bimetallic [NiFe]-hydrogenase is responsible for the reversible oxidation of di­hydrogen (Lubitz *et al.*, 2014 ▸); the diiron center in methane monoxygenases enables methane conversion in bacteria (Jacobs *et al.*, 2021 ▸); and complex FeS clusters facilitate electron transfer (Lauterbach *et al.*, 2016 ▸), metabolic processes (Gee *et al.*, 2021 ▸) and can play pivotal structural roles in proteins (Kennepohl & Solomon, 2003 ▸). The pinnacle of these polyatomic clusters is arguably the Mo_1_Fe_7_S_9_ cluster found in the di­nitro­gen-reducing enzyme nitro­genase, which is proposed to conduct its reaction through an eight-step cycle where the enzyme mediates the introduction of protons, electrons and H_2_ evolution to form ammonia from di­nitro­gen (Lowe & Thorneley, 1984 ▸). Note that nearly all of the metalloenzyme systems described so far have been researched by Keith Hodgson and/or scientists trained by Hodgson, thus his impact to this field cannot be overstated.

Metalloproteins played a key role in the history of X-ray crystallography. Structural biological applications of monochromatic synchrotron radiation were reported in 1976 by Hodgson’s team with results from the Stanford Synchrotron Radiation Project, later known as Stanford Synchrotron Radiation Laboratory, and today known as the SSRL. These data also included the first synchrotron diffraction images of metalloproteins: rubredoxin and azurin (Phillips *et al.*, 1976 ▸; Phillips *et al.*, 1977 ▸).

The application of synchrotron radiation towards metalloprotein structural biology rapidly grew in subsequent decades. Bright tuneable sources allowed utilizing the metal X-ray absorption edges themselves as a method to solve phases via anomalous scattering (Phillips & Hodgson, 1980 ▸). Despite these advances, it became evident that lightsource enhancements to achieve high-resolution structures was incongruous with the integrity of metalloproteins that, by their very nature, leverage the sensitive electronic structure of transition metals. Deleterious effects from synchrotron radiation on protein structures, specifically the local coordination spheres of the critical metallocofactors, have been a growing challenge and correlate with the increasing average flux of upgrading synchrotrons (Ravelli & Garman, 2006 ▸).

Radiation damage control approaches were developed, such as cryogenic cooling of crystals (Garman & Schneider, 1997 ▸), dose-mitigation strategies (Bourenkov & Popov, 2010 ▸; Zeldin *et al.*, 2013 ▸) and multi-modal optical spectroscopic monitoring (Cohen *et al.*, 2016 ▸). These strategies work well for metalloproteins as their cofactors generally confer strong optical absorbances reporting on X-ray-induced damage. A hallmark example of this latter approach was utilized for the enzyme intermediate Horseradish Peroxidase Compound-I, which contains an Fe(IV)=O moiety with a radical cation hole in the porphyrin macrocycle (Thomas, 1993 ▸). In this system, careful monitoring of the heme optical *Q*-bands was critical to producing the first reliable structure of this intermediate (Meharenna *et al.*, 2010 ▸), which was previously highly contested (Behan & Green, 2006 ▸). Another system with a notable discrepancy was that of the Mn_4_Ca cluster in the oxygen-evolving complex of Photosystem II, where synchrotron structures (Zouni *et al.*, 2001 ▸; Kamiya & Shen, 2003 ▸; Biesiadka *et al.*, 2004 ▸; Ferreira *et al.*, 2004 ▸; Kulik *et al.*, 2007 ▸) mapped intermetallic distances that were at odds with multiple spectroscopies (Yachandra *et al.*, 1996 ▸; Cinco *et al.*, 2004 ▸; Peloquin *et al.*, 2000 ▸), indicating radiation damage may be the source of these inconsistencies (Yano *et al.*, 2005 ▸).

To manage the radiation-damage issues without the trade-offs of lost spatial resolution, X-ray pulse intensities that temporally probe the crystal before its degradation are required (Neutze *et al.*, 2000 ▸). These fluxes and pulse lengths became available with the development of XFELs (Chapman *et al.*, 2011 ▸; Seibert *et al.*, 2011 ▸). These innovations, now collectively called femtosecond crystallography, also allowed for the measurement of crystals under ambient conditions, making structures better reflect the conditions in which the proteins operate. With the capabilities in place, the first metalloenezyme structure measured at the LCLS XFEL was Photosystem I which contained a series of Fe_4_S_4_ clusters as part of a light-driven electron transport chain (Chapman *et al.*, 2011 ▸).

A renaissance of SFX-driven structural biology soon followed, and with it, a boon to research on metalloproteins. Metalloenzymes were some of the early candidates for XFEL crystallography experiments either for validation or because they were not amenable to any conventional methods. Various forms of liquid jets were first utilized to deliver crystals and some of the first metalloprotein XFEL-derived structures that followed were of the Zn-containing thermolysin protease (Hattne *et al.*, 2014 ▸), ferredoxin [4Fe4S] electron transfer protein (Nass *et al.*, 2015 ▸), the purple bacteria reaction center (Johansson *et al.*, 2013 ▸), photosystem I (Aquila *et al.*, 2012 ▸) and photosystem II (Kern *et al.*, 2014 ▸).

Additionally, fixed-target methods persisted into the XFEL structural biology era with a key study demonstrating XFEL determination of metalloenzyme structures using large crystals fixed on a goniometer to measure the structure of myoglobin and [FeFe]-hydrogenase (Cohen *et al.*, 2014 ▸). Another contemporaneous study at SACLA demonstrated the first undamaged structure of the oxidized heme–copper enzyme cytochrome c oxidase (Hirata *et al.*, 2014 ▸).

In addition to these pioneering experiments, metalloproteins were crucial in confirming that the brief XFEL pulses accurately captured the structures and metallocofactors before any damage or alteration to the protein structure occurred. Due to the sensitive electronic structure afforded by metallocofactors, metalloenyzmyes are a few orders of magnitude more sensitive at the cofactor to radiation damage than other enzymes in their total structures (Yano *et al.*, 2005 ▸).

The electronic structure of the Fe_4_S_4_ cluster of ferredoxin was utilized as a surrogate probe of the damage by exposing the sample to high power densities afforded by the nanofocus capabilites at CXI (Nass *et al.*, 2015 ▸) as it could be monitored by Fe X-ray emission spectroscopy (XES). This work and others (Chapman, 2015 ▸) led to determinations that the majority of scattered signals originate from undamaged samples based on the nominal pulse intensities and pulse lengths of time.

**Figure 3:**
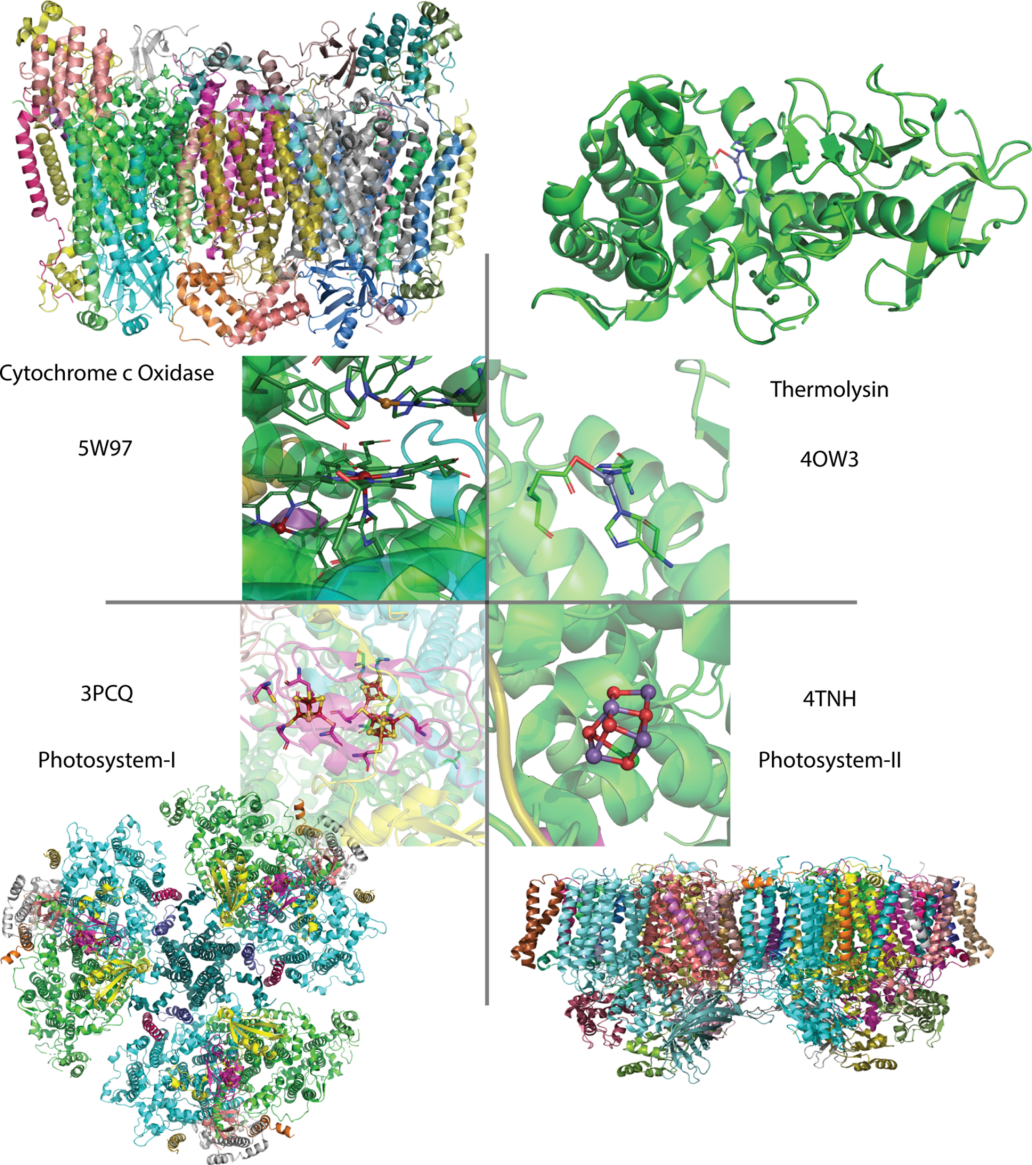
Highlights of various metalloprotein structures obtained at the LCLS, where their radiation-sensitive cofactors made them amenable targets for SFX rather than conventional synchrotron approaches. Insets show their key active site cofactors. Insetted text includes names and PDB codes for the structures which correspond to cytochrome c oxidase (Ishigami *et al.*, 2017 ▸), thermolysin (Hattne *et al.*, 2014 ▸), photosystem I (Chapman *et al.*, 2011 ▸) and photosystemII (Kern *et al.*, 2012 ▸).

Owing to their unique electronic and geometric structures, inherent sensitivity to radiation damage, and rich chemistry, metalloenzymes remain a key target for XFEL experiments in the biological sciences. Fig. 3 ▸ highlights some of the metalloprotein structures obtained at LCLS.

### 5.3. Structural dynamics

In addition to elucidating the structure of challenging classes of proteins, such as membrane proteins and metalloenzymes, the SFX method allows both intrinsic and triggered macromolecular dynamics to be captured. Time-resolved SFX (TR-SFX) experiments rely on a trigger that is applied to the crystal at a defined time delay before the arrival of the X-ray probe. The vast majority of TR-SFX experiments make use of an optical laser pulse to trigger and synchronize protein dynamics (Fig. 1 ▸), which has found extensive use in the study of photosensitive proteins (Kern *et al.*, 2013 ▸). While the first experiments recorded the atomic rearrangements on a millisecond timescale, a concerted effort followed to push TR studies into the femtosecond time domain (Tenboer *et al.*, 2014 ▸). By making use of femtosecond optical lasers and the unique, ultrafast XFEL pulses, some of the fastest chemical processes in biology have been observed, such as the light-triggered dissociation of carbon monoxide from myoglobin (Barends *et al.*, 2015 ▸), or the *cis*–*trans* isomerization in fluorescent proteins (Pande *et al.*, 2016 ▸; Hosseinizadeh *et al.*, 2021 ▸; Fadini *et al.*, 2023 ▸) and retinal-bound ion transporters (Nogly *et al.*, 2018 ▸; Nass Kovacs *et al.*, 2019 ▸).

**Figure 4:**
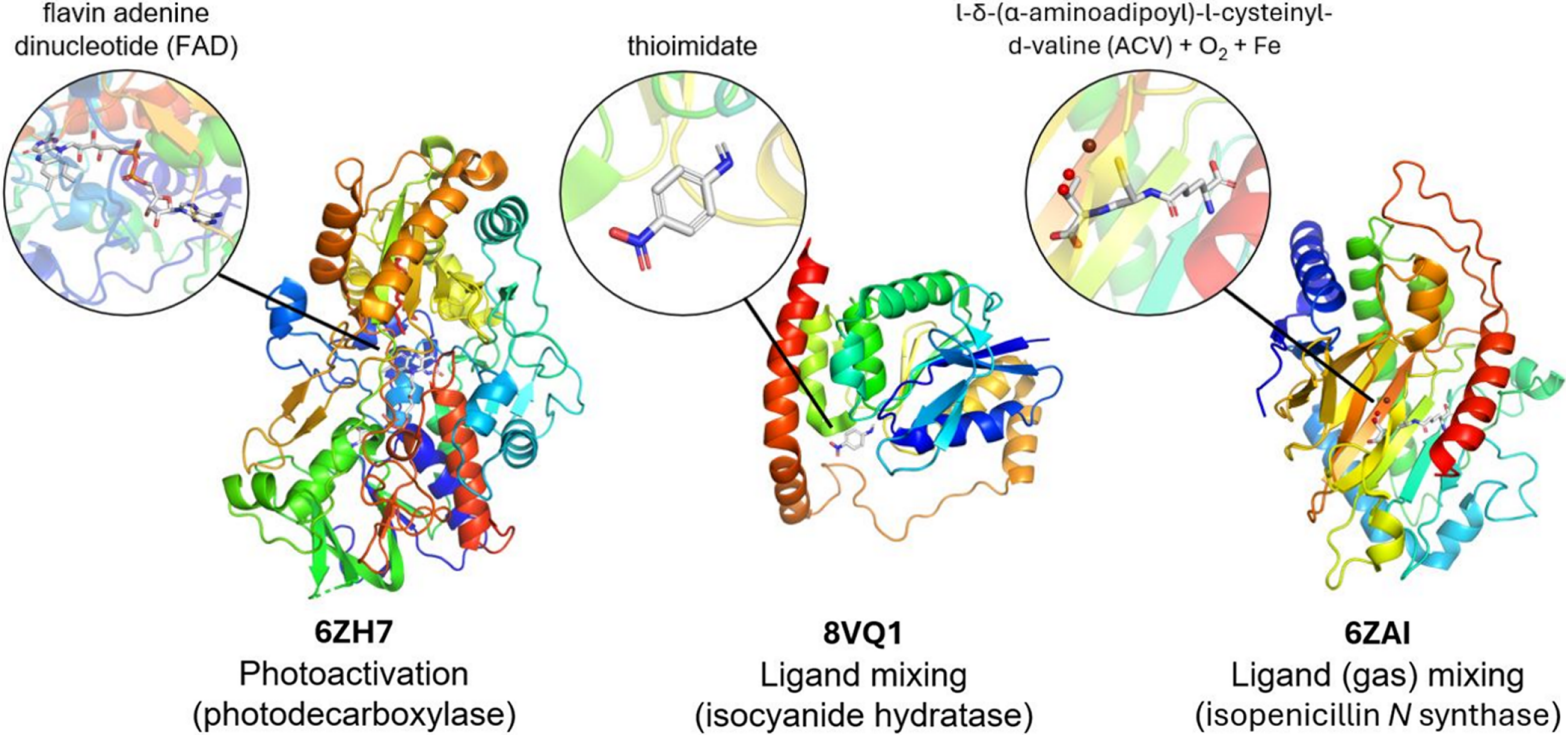
Highlights of various systems studied using TR-SFX. This method has been used at the LCLS to study light-driven sensors, ion transport (*e.g.* by bacteriorhodopsin, see Fig. 2 ▸) and photoenzymes [*e.g.* photode­carboxyl­ase (Sorigué *et al.*, 2021 ▸)]. To study the dynamics of enzymes that are not inherently photosensitive, alternative methods exist to trigger conformational change, such as ligand (Smith *et al.*, 2024 ▸) or gas mixing (Rabe *et al.*, 2021 ▸). PDB codes are displayed underneath the structures.

Although TR-SFX experiments initially relied on optical laser pumping, continued developments in the field have now resulted in a wide range of available triggering methods available at LCLS and other X-ray light sources. Considering that less than 0.5% of proteins are estimated to be naturally photosensitive (Monteiro *et al.*, 2021 ▸), these developments have greatly expanded the range of biological systems to which the TR-SFX method can be applied (Fig. 4 ▸). One of the most successful methods for studying the dynamics in non-photosensitive proteins relies on rapid mixing (‘mix-and-inject’) of enzymes with a ligand (Stagno *et al.*, 2017 ▸) or gas (Srinivas *et al.*, 2020 ▸; Rabe *et al.*, 2021 ▸). While mixing is applicable to a wide range of systems, the dynamics are typically limited by the diffusion rate to the millisecond time domain. Alternatively, crystals can be soaked or co-crystallized with photocages to trigger dynamics in systems using a blue light or UV pulse. Examples include the TR studies of the ATP-driven dimerization of the MsbA nucleotide-binding domain (Josts *et al.*, 2018 ▸), allosteric motions in the fluoro­acetate dehalogenase FAcD (Mehrabi *et al.*, 2019 ▸) and hydride transfer in an NO reductase (Tosha *et al.*, 2017 ▸; Nomura *et al.*, 2021 ▸). Next to releasing a ligand, such photocages can be used to release an acid or base to rapidly alter the pH following a pump laser pulse (Monteiro *et al.*, 2021 ▸). Similar to photocaged compounds, protein dynamics of non-photosensitive systems have been studied through the incorporation of photoswitches in the crystal. Photocages rely on a photo-induced cleavage of a covalent bond whereas photoswitches undergo a reversible isomerization reaction on exposure to UV or visible light. A recent study reporting the structural dynamics of tubulin following the release of a photoswitchable anti-cancer compound (Wranik *et al.*, 2023 ▸) demonstrates that novel triggering methods not only benefit the TR-SFX method, but also the development of biomedical applications. Finally, other novel, universally applicable triggering methods currently under development include laser-induced temperature jumps through infrared (IR) laser pumping (Thompson *et al.*, 2019 ▸; Wolff *et al.*, 2023 ▸) which may extend into the terahertz regime in the future (Lundholm *et al.*, 2015 ▸), and electric field crystallography (Hekstra *et al.*, 2016 ▸).

Note that even the most mature experimental setups for performing TR serial crystallography experiments are still undergoing rapid developments and improvements. For example, the field has recently started to recognize the dependence of the structural dynamics on the pump laser fluence used in optical pump–probe experiments (Barends *et al.*, 2024 ▸). Furthermore, elaborate pump–probe schemes have demonstrated the contribution of vibrational and electronic processes to the conformational dynamics in ultrafast TR experiments (Hutchison *et al.*, 2023 ▸). Such studies provide a clear path forward towards improved TR experiments and an improved understanding of ultrafast structural dynamics in biology.

Following the interest in TR crystallography at the XFEL, similar setups have been successfully used for obtaining room-temperature (Weinert *et al.*, 2017 ▸) or TR structures at the synchrotron. The TR-SSX method has enabled the observation of molecular motion on a millisecond timescale (Schulz *et al.*, 2018 ▸; Weinert *et al.*, 2019 ▸). The combination of TR-SFX and TR-SSX provides a comprehensive understanding of macromolecular dynamics from the ultrafast atomic motion (SFX) through larger conformational changes in the secondary protein structure on longer timescales (SSX) (Mous *et al.*, 2022 ▸). The synergy between the SFX and SSX techniques, together with the opportunity to collect corroborative data for XFEL experiments (Lin *et al.*, 2024 ▸) highlight the benefit of having a synchrotron light source located near the XFEL.

Although most studies of the structural dynamics in macromolecules at LCLS make use of the TR-SFX method, a similar experimental geometry can be used to collect dynamic information on biomolecules in solution using TR-WAXS. Similar to TR-SFX experiments, TR X-ray scattering can make use of optical laser pumps (Arnlund *et al.*, 2014 ▸) or ultrafast liquid mixers (Zielinski *et al.*, 2023 ▸) to capture conformational dynamics over a wide time range. The extreme brightness of the LCLS allows the measurement of the scattering signal from micrometre-scale volumes, enabling homogeneous light excitation in optical pump–probe experiments and rapid diffusion during mixing experiments.

## 6. Current LCLS capabilities in structural biology

LCLS utilizes three primary methods for structural biology: (1) macromolecular crystallography (MX), (2) solution scattering like small- and wide-angle X-ray scattering (SAXS/WAXS), and (3) single particle imaging (SPI). As of 2024, most structural biology experiments involve MX using SFX techniques. These techniques are relatively insensitive to the chemical (or redox) state of elements, so SFX is often paired with X-ray emission spectroscopy (XES) to track both the geometric and the chemical states of important elements, such as transition metals. The structural biology program at the LCLS is primarily hosted at two instruments: CXI and MFX, where developments in experimental capabilities have focused on delivering biological samples to X-rays in a physiologically relevant state and effectively triggering them to observe subsequent structural changes. This includes methods for triggering reactions, such as light triggering with lasers or chemical triggering using mixing nozzles.

### 6.1. Instruments overview

This section provides an overview of the key instruments and methodologies supporting structural biology at LCLS, highlighting their unique capabilities and applications. Specifically, we highlight the in vacuum CXI instrument where a very low background enables highly sensitive experiments and the in-air MFX instrument which benefits from substantial experimental flexibility. We also discuss advanced sample delivery methods that ensure precise and efficient interaction with the X-ray beam. Each of these instruments and techniques plays a pivotal role in pushing the boundaries of structural biology and enhancing our understanding of complex biological systems.

#### 6.1.1. MFX instrument

The MFX instrument at LCLS, commissioned in 2016, was designed to accommodate the demand for SFX experiments (White *et al.*, 2015 ▸). MFX offers a versatile in-air endstation table that allows for various sample delivery methods, experimental enclosures and geometries, providing flexibility that complements the *in vacuo* capabilities of the CXI instrument.

The LCLS XFEL beam is directed to the MFX line via a hard X-ray mirror located in the X-ray transport tunnel (XRT), which deflects the beam by −5.5 mrad relative to the CXI mainline. Beamline components such as stoppers and attenuators are positioned upstream of the hutch on a diagnostic stand. This stand also houses a pulse-picker, allowing the 120 Hz beam to be downsampled to 30 Hz (or lower) to match the readout rates of some detectors, for example the MFX Rayonix MX340-XFEL detector (Armenta & Paiser, 2018 ▸), or to better match the sample replenishing rates of various sample delivery methods.

Inside the hutch, two stands are equipped with various diagnostic beam interfaces. Intensity-position monitors (IPMs) utilize SiN targets of varying thicknesses to backscatter the X-rays onto diodes arranged around the beam axis, providing data on beam flux and position. Profile intensity monitors use cameras to observe YAG screens that fluoresce when struck by X-rays, giving insight into the beam’s spatial profile.

The beam is focused at MFX using ten sets of Be compound refractive lenses (CRLs) (Snigirev *et al.*, 1996 ▸) housed in a vacuum chamber as part of the transfocator system (TFS) (Vaughan *et al.*, 2011 ▸). Despite their chromatic focusing properties, the CRLs can be adjusted by varying lens combinations and translating the system within a 300 mm range along the beam, enabling the focusing of a broad range of energies with minimal coverage gaps at the nominal X-ray-to-sample interaction point (IP). The standard configuration of the TFS typically operates within an 8–16 keV range, focusing the beam down to 2–3 µm at the IP.

**Figure 5:**
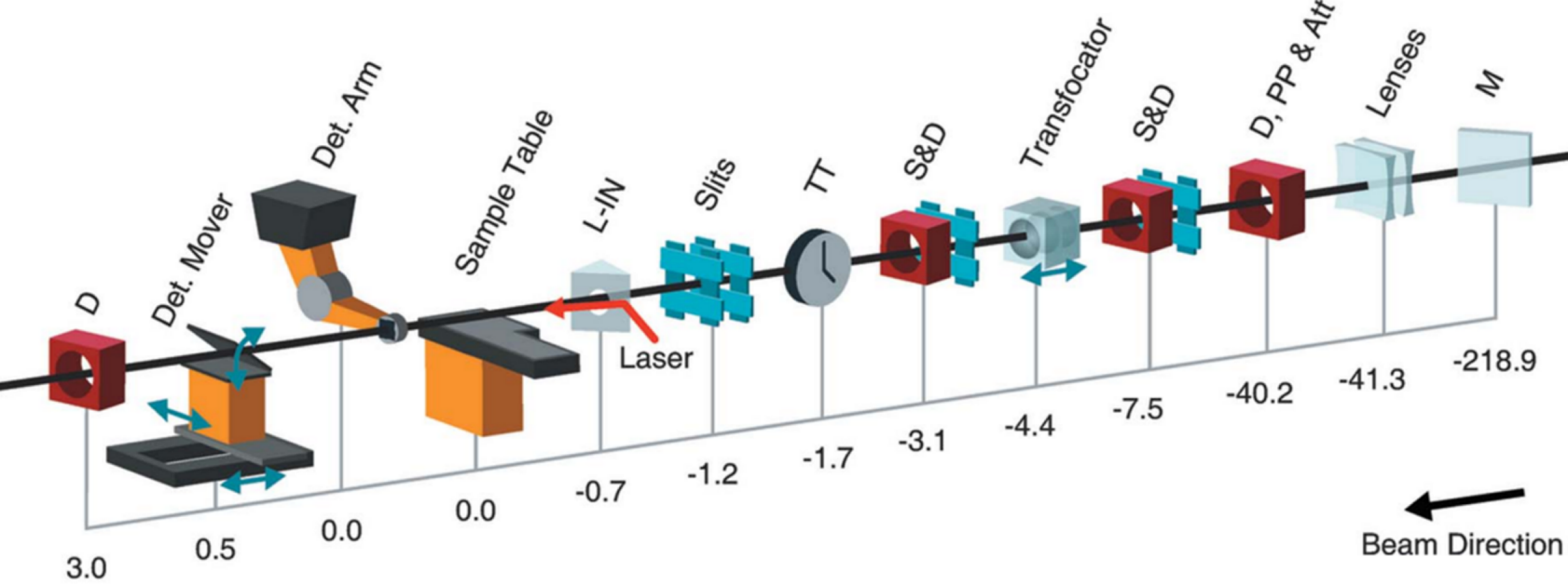
Overview of the MFX instrument layout. Distances are in meters from the nominal interaction region on the sample table; positive values indicate the beam’s propagation direction. M is a mirror in the LCLS XRT for harmonic rejection and beam deflection to MFX. Lenses at 41.3 are pre-focusing CRLs. D, PP and Att at 40.2 is a diagnostic section with a Ce:YAG screen, single-pulse-picker and ten silicon attenuators. S and D are slits and diagnostics for beam-viewing and intensity measurement. A transfocator mounts the CRLs for a controllable spot size. A TT, now known as an arrival time monitor, measures the optical laser’s arrival time relative to the X-rays. The double slits at 1.2 block beam harmonics. L-IN is the laser in-coupling for the optical laser. D at 3.0 is a Ce:YAG screen diagnostic to view the post-detector beam. The MFX sample (0.0 on this figure) is about 420 m downstream of the X-ray source within the undulator. Reproduced with permission from Sierra *et al.* (2019 ▸).

The final key component in the beamline is the arrival time monitor (labeled TT for ‘time tool’ in Fig. 5 ▸), which corrects for timing jitter on a shot-to-shot basis, essential for ultrafast optical pump X-ray probe experiments (Bionta *et al.*, 2011 ▸; Bionta *et al.*, 2014 ▸; Harmand *et al.*, 2013 ▸).

The primary beamline terminates through a diamond window, typically followed by a He flight tube leading to the sample table. Various endstation configurations can be deployed on this table.

For optical–pump experiments, the beam often initially passes through the laser in-coupling box (LIB) on the table, which integrates an optical excitation laser orthogonally to the incident beam. A holey mirror within the LIB aligns the two beams nearly colinearly. Insertable diodes and a Ti scattering foil within the LIB provide timing diagnostics. If the experimental setup or wavelength (*e.g.* near-IR) is incompatible with the LIB, laser coupling is performed instead with optomechanics directly on the sample table.

Endstations at MFX typically feature various sample delivery methods, including liquid jets, high viscosity extrusions, droplets or fixed-target holders. These setups often include at least three axes of visualization for the sample as it interacts with the beam. To minimize photon loss in air, the sample environment can be enclosed in He when compatible, or all photon paths can be bridged by He-filled flight tubes or windowed ‘cones’, typically for XES measurements.

After the sample location, a large format area detector collects the scattering/diffraction signals. At MFX there are two detector options: a robotic arm above the X-ray/sample interaction point can move an ePix10k2m (van Driel *et al.*, 2020 ▸) into position or a downstream detector mover stage can move a Rayonix MX340-XFEL into the post-sample position. The core trade-offs between the two configurations are active sensing area, dynamic range and accessible repetition rates that are then delivered via the pulse-picker (described above).

#### 6.1.2. CXI instrument

**Figure 6:**
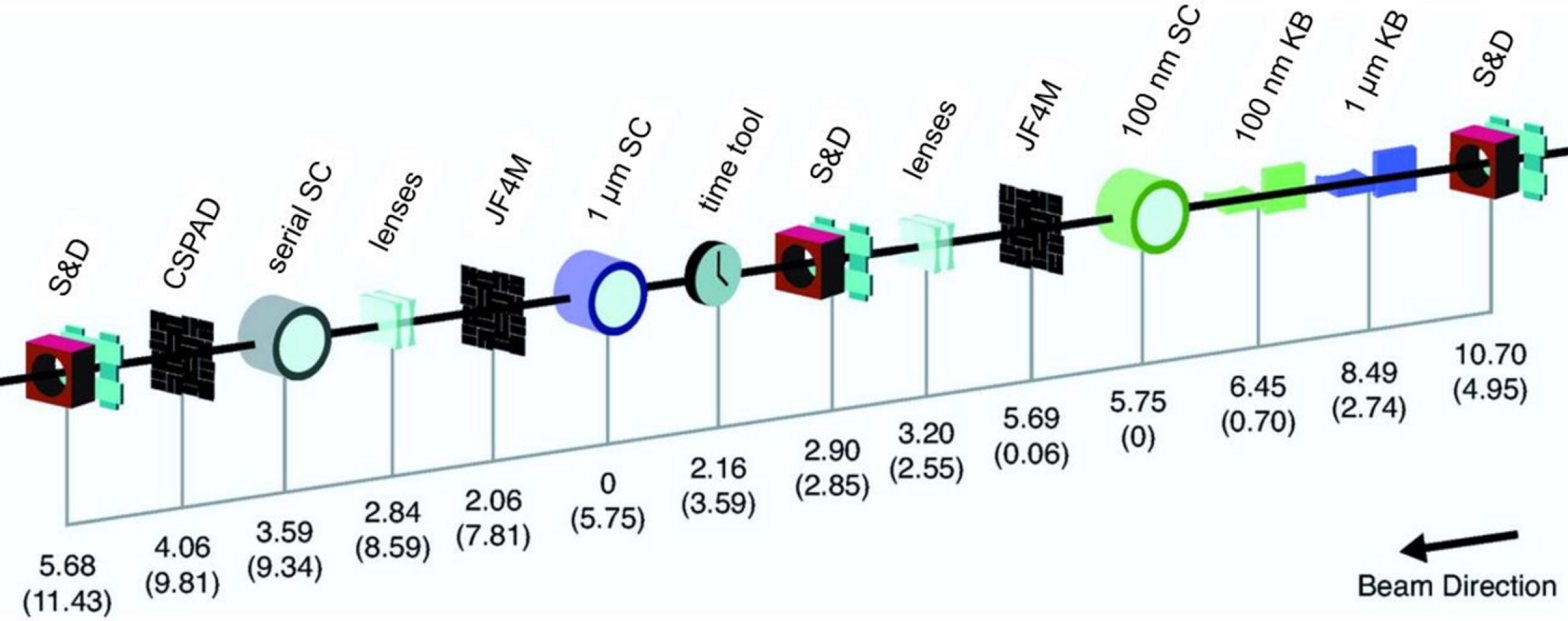
Overview of the CXI instrument layout. Distances are in meters from the interaction point in sample chamber 1, SC1 (or the interaction point in sample chamber 2, SC2, in brackets). X-rays are focused into the SC1 chamber using the 1 µm Kirkpatrick–Baez (KB) mirrors, and into the SC2 chamber using the KB2 mirrors. X-rays can be refocused downstream of the SC1 chamber using a set of lenses for a serial experiment in the serial SC. S and D are slits and diagnostics for beam-viewing and intensity measurements. The TT measures the optical laser’s arrival relative to the X-rays in pump–probe experiments. The JUNGFRAU 4M (JF4M) detector can be flexibly placed either downstream of the SC1 or SC2 chamber. The serial sample chamber (serial SC) is paired with the CSPAD 2.3M detector or an ePix10k module. Reproduced with permission from Liang *et al.* (2015 ▸) and modified to reflect the latest beamline configuration.

The CXI endstation of LCLS is an in-vacuum instrument specializing in forward-scattering experiments. Though the vacuum environment of CXI provides more restraints to the experiment than MFX, the low background scattering signal makes the instrument an ideal option for weakly scattering systems (*e.g.* nanocrystals). The instrument is situated on the direct line coming from the XRT and hosts three different interaction points in its hutch, enhancing the instrument’s versatility. The main CXI chamber is sample chamber 1 (SC1), in which the beam is focused to a 1.3 µm × 1.3 µm spot size using the KB1 mirror system, and most of the SFX experiments are performed. SC1 is complemented by sample chamber 2 (SC2, sometimes referred to as SC01), which offers an exceptionally high X-ray fluence of up to 10^20^ W cm^−2^ by focusing the FEL beam down to 90 nm × 150 nm using a separate, KB2 mirror system. The exceptionally high fluence makes the SC2 chamber suitable for coherent diffractive imaging of single particles. Both CXI KB mirror systems can be fully retracted from the beampath, allowing switching between the micro- and nanofocus configurations. The long focal length for the KB1 mirrors (8.7 and 8.3 m for the horizontal and vertical focusing mirrors, respectively) allow the high-curvature KB2 mirrors (0.9 and 0.5 m focal length for the horizontal and vertical focusing mirrors, respectively) and SC2 sample chamber to be placed in between the KB1 mirror system and the SC1 chamber (Fig. 6 ▸). The reflectivity cutoff for the KB1 mirrors is 11 keV and, therefore, two stacks of inline Be CRLs are available for producing a 3–50 µm focus at higher photon energies (Liang *et al.*, 2015 ▸).

The third sample chamber SC3, also referred to as the serial sample chamber (SSC), is located closely downstream of SC1, and allows the use of the spent beam coming from the SC1 chamber (Boutet *et al.*, 2015 ▸). Through the use of an additional stack of Be CRLs, the beam exiting the SC1 chamber can be refocused to a <10 µm spot size. This enables a second, parasitic experiment to take place in parallel with the main experiment in the SC1 chamber. When SSC is not in use for parasitic operation, the chamber can be used for a second in-vacuum downstream detector to capture SAXS signal passing through the flight tube of the primary detector.

Further versatility of the CXI instrument is offered through flexible sample and detector geometries. The main forward X-ray scattering detector used at CXI is the JUNGFRAU 4M (Mozzanica *et al.*, 2018 ▸), which replaced the CSPAD 2.3M detector (Hart *et al.*, 2012 ▸) in 2020. In addition, a compact ePix100 (Carini *et al.*, 2016 ▸) and ePix10k camera (Blaj *et al.*, 2019 ▸) can be flexibly placed to detect emission lines in XES experiments or a SAXS signal in SC3. With regards to sample delivery, samples can be delivered to the interaction point using either liquid jets [including gas-dynamic virtual nozzles (GDVNs), dual flow focusing nozzles and mixing injectors], high viscosity extruders or samples on a fixed target. Further details regarding sample delivery modalities are provided in the Methods[Sec sec6.2].

Methods that the instrument caters to include primarily experiments that are highly sensitive to the signal-to-noise of the forward scattering signal, such as TR-WAXS (Zielinski *et al.*, 2023 ▸) and diffraction experiments on nanocrystals obtained through *in vivo* crystallization (Tetreau *et al.*, 2022 ▸). In addition, by making use of the free space femtosecond laser setup at CXI, it is possible to capture the small difference signals in ultrafast optical pump–probe SFX experiments (Nogly *et al.*, 2018 ▸; Hosseinizadeh *et al.*, 2021 ▸).

### 6.2. Methods

The high intensity of the LCLS pulses makes conventional crystallography and solution scattering methods challenging, if not impossible, to implement robustly because biological samples generally do not survive an exposure to an X-ray pulse from LCLS. Consequently, methods of serial data collection were developed in which a new sample was introduced to the LCLS interaction point between the arrival of separate pulses. The method of serial crystallography was developed to deliver protein crystals to LCLS in a way that enabled high-resolution, low-background crystallography data.

#### 6.2.1. Sample delivery

A critical aspect to the success of structural biology experiments at LCLS is effectively delivering the sample to the X-ray interaction point in a way that preserves the state of the sample for the data collection. A key divergence of sample delivery for biology at XFELs is that the short pulse duration allows measurements to be made under physiological conditions and avoids the need for cryogenic cooling. This ability to take data on samples that are not cryogenically cooled has a significant upside in the quality of data that can be collected in that the data will be more physiologically relevant, but come with the significant challenge that, unlike the case of cryogenically frozen samples, the physical chemical properties are not dominated by the formation of vitreous ice. Practically, this means that fluid properties for samples and buffers, such as viscosity and surface tension, will have a direct impact on how these samples can be transported to the interaction point without compromising the sample itself. Due to the diversity in the samples and buffers for biological sciences, quite a diverse suite of sample delivery techniques have been developed depending on sample volume, viscosities, surface tension, hydro­phobicity *etc*.

The original liquid jet used for serial experiments at LCLS was developed to deliver very small samples, potentially as small as fully hydrated single particles, to advanced X-ray and electron sources sources. This liquid jet, referred to as the GDVN (DePonte *et al.*, 2008 ▸; Gañán-Calvo, 1998 ▸) uses high-speed gas to produce a thin liquid jet from an aperture and became the original workhorse for SFX crystallography at LCLS (Chapman *et al.*, 2011 ▸). At a repetition rate of 120 Hz, the GDVN requires large sample quantities to collect a complete dataset and considerable effort was put into alternatives for serial measurements that would reduce sample consumption. Additionally, due to the nature of the jet formation in the GDVN, higher-viscosity samples are difficult/impossible to inject and so efforts to extend serial measurements to high-viscosity samples, such as those containing membrane proteins, were pursued.

High-viscosity extruders such as the LCP injector (Liu *et al.*, 2013 ▸) and the grease injector (Shimazu *et al.*, 2019 ▸) enabled the study of samples in high-viscosity media. The LCP injector was developed to deliver membrane protein crystals grown in the LCP crystallization medium by having a large pressure-amplification near the sample reservoirs (Liu *et al.*, 2013 ▸). The LCP and high-viscous media still flow fast enough to enable TR experiments driven by optical lasers (Nogly *et al.*, 2015 ▸; Mous *et al.*, 2022 ▸) at lower repetition rates.

Sample consumption was the biggest issue initially and reducing sample consumption was approached through several different methods, including embedding samples in high-viscosity media (Sugahara *et al.*, 2020 ▸; Sugahara *et al.*, 2015 ▸; Conrad *et al.*, 2015 ▸; Martin-Garcia *et al.*, 2017 ▸; Shimazu *et al.*, 2019 ▸); electro-kinetically forming a thin jet (Sierra *et al.*, 2012 ▸); hydro­dynamically focusing samples using an immiscible sheath or co-flow liquid (Sierra *et al.*, 2015 ▸; Oberthuer *et al.*, 2017 ▸); generating sample droplets synchronized in time with the XFEL with free space propagation (Roessler *et al.*, 2013 ▸; Roessler *et al.*, 2016 ▸; Su *et al.*, 2021 ▸), delivered via moving substrate (called the ‘tape drive’) (Fuller *et al.*, 2017 ▸) or embedded in a co-flow liquid (Doppler *et al.*, 2023 ▸; Sonker *et al.*, 2022 ▸); and using a fixed target that is rapidly scanned through the interaction point (Hunter *et al.*, 2014 ▸; Frank *et al.*, 2014 ▸; Cohen *et al.*, 2014 ▸; Roedig *et al.*, 2015 ▸).

An important realization was that, for hydro­dynamically focused injectors, if the sheath is miscible with the sample, then hydro­dynamic focusing will be replaced by mixing of the two liquids (Wang *et al.*, 2014 ▸), which can be used for TR chemical triggering of samples such as enzymes (Schmidt & Saldin, 2014 ▸) in a process called mix and inject serial crystallography (Kupitz *et al.*, 2017 ▸; Olmos *et al.*, 2018 ▸; Dasgupta *et al.*, 2019 ▸).

## 7. Future plans

LCLS is always developing and pushing the boundaries of what is possible in biological and physical science research. LCLS-II affords a new high repetition rate X-ray source with the LCLS-II-HE upgrade to bring this source to the hard X-ray regime. Concomitant with this development on the high-energy regime, future enhancements will focus on several key areas: the upgrade of beam transport, diagnostics and instrument devices at the hard X-ray beamlines; the integration of high repetition rate laser systems; and the capacity to handle a higher average X-ray power. These upgrades are designed to enable more detailed sampling of dynamic processes, facilitate nonlinear spectroscopies and substantially increase throughput of user science at the facility.

This section explores how these developments will transform the existing biological sciences instruments into the advanced MFX-HE and CXI-HE. The future trajectory includes higher-rate X-ray detectors, optimized X-ray optics, improved sample delivery methods and complex data analysis tools to handle the high repetition rate, all of which aim to enhance experimental capabilities with a high-repetition rate X-ray beam.

### 7.1. LCLS-II and LCLS-II-HE capabilities

The Linac Coherent Light Source II (LCLS-II) project significantly expanded the capabilities of the LCLS facility by introducing a superconducting accelerator along SLAC’s existing linear accelerator tunnel. Unlike the copper linac of LCLS-I, which operates at a maximum repetition rate of 120 Hz, the superconducting linac of LCLS-II is capable of delivering pulses at rates up to 1 MHz while maintaining comparable LCLS-I shot-to-shot pulse energies when operating within tens of kilohertz. This substantial increase in pulse frequency and average power opens new opportunities for TR experiments, particularly those requiring high signal throughput, such as studies of ultrafast dynamics in materials and dilute biological systems.

The current high repetition-rate X-ray beam generated by LCLS-II consists of evenly spaced pulses with variable energy across the soft X-ray regime, which ranges from 200 to 1300 eV. The electron injection for this system is achieved using a 186 MHz continuous wave radio-frequency gun (Doyle *et al.*, 2017 ▸). The electron acceleration is carried out through 37 individual supercooled microwave cavities known as cryomodules that bring the electron beam energy up to 4 GeV (Solyak *et al.*, 2018 ▸).

For SASE generation, LCLS-II primarily utilizes the soft X-ray undulators. The electron beam is directed into newly designed beam dumps (Santana-Leitner *et al.*, 2014 ▸), while the X-ray pulses continue to the experimental halls. Here, it can be utilized at the TMO, ChemRIXS or qRIXS instruments, enabling a broad range of cutting-edge scientific experiments in the soft X-ray regime.

The high-energy upgrade project (LCLS-II-HE) will provide an expansion and enhancement of the LCLS-II capabilities by extending the existing LCLS-II accelerator to achieve electron beam energies greater than 8 GeV which will enable photon energies up to 13 keV at repetition rates up to 1 MHz.

To accommodate these upgrades, the beam transport, diagnostics and instrument devices at the hard X-ray beamlines (XPP, MFX, XCS/DXS, CXI) will be upgraded to accommodate the higher average power. As mentioned below, the higher repetition rate also coincides with an analogous increase in data generation and throughput which will require analogous upgrades to the data infrastructures. The upgrades to the instruments for LCLS-II-HE will maintain compatibility to utilize the 120 Hz copper linac beam which affords operational flexibility between the two accelerators. New laser systems and spaces will be incorporated into the experimental halls that can accommodate delivery of high repetition rate optical beams to match the X-ray delivery. Collectively, these developments will enable nonlinear spectroscopies that benefit from the high average power, higher sampling of dynamic processes in time or structural domains, and increased throughput of user science at the facility. The latter two benefits will have an immediate enhancement on biological science research at the LCLS.

### 7.2. Future instruments and beamlines

The majority of bioscience experiments following the commissioning of LCLS-II-HE is expected to take place at the MFX-HE instrument. The instrument will make use of the increased repetition rate through an upgrade to high rate X-ray detectors. Initially, an ePixUHR, capable of a 35 kHz frame rate with 4 megapixels will be available. In addition to the upgrades to the X-ray detectors, the X-ray optics for the MFX instrument will be upgraded from Be CRLs to KB mirrors to improve the X-ray focusing down to 1 µm × 1 µm. Together, these upgrades will make MFX-HE the flagship instrument for high-throughput SFX and XES experiments.

The CXI-HE instrument will undergo a considerable upgrade and offer new chambers and X-ray optics for all interaction points. Similar to the LCLS-I CXI instrument, an in-vacuum 100 nm and 1 µm focus chamber (referred to as IP1 and IP2) will be offered. The design for liquid jet experiments in IP2 will mimic the liquid jet configuration currently available at the LCLS-II ChemRIXS instrument. This includes the design for the load-lock system for sample exchanges and a sample recirculation system. The liquid jet chamber will be compatible with both vertical and horizontal injection, enabling simultaneous collection of XRD and XES data. The KB system will be upgraded with bendable mirrors, which will enable X-ray focusing at higher photon energies than is currently possible at the CXI instrument. The SC3 sample chamber, used for performing experiments with the spent beam coming from the SC1 chamber, will no longer be available at the CXI-HE instrument. Instead, a new setup for ambient X-ray solution scattering and XES experiments will become available (IP3). The configuration will be similar to the condensed phase chemistry configuration currently offered at the XCS instrument.

In addition to the future MFX-HE and CXI-HE instruments, the tender X-ray instrument (TXI) is expected to have a significant impact in biosciences in the coming years. The TXI instrument will serve three scientific areas: non-linear X-ray science through X-ray pump and X-ray probe techniques (through combining X-ray pulses from the soft and hard X-ray undulators), tender X-ray spectroscopy (including X-ray absorption and emission spectroscopy), and coherent X-ray imaging.

Tender X-ray spectroscopy has exciting applications in the biological sciences, as the *K*-edge of the elements P, S, Cl, K and Ca are located in the 2–5 keV tender X-ray regime. Therefore, the tender X-ray spectroscopy setup of TXI can be used to capture the electronic state of sulfur in, for example, cysteines, including thiols, di­sulfides and oxidized species that have been proposed to modulate protein function (Lo Conte & Carroll, 2013 ▸). Many other biomolecules critical to enzymatic catalysis such as ATP and NADPH can be investigated, including hydrolysis, hydrogen bonding or electrostatic effects (Mathe *et al.*, 2021 ▸). Compared with synchrotron-based tender X-ray spectroscopy techniques, the extremely high average flux of LCLS-II is expected to provide distinct advantages and enable the signal to be measured from samples at low concentrations (at or below millimolar concentrations) and active state populations created through laser pumping.

In addition to tender X-ray spectroscopy, TXI is expected to be instrumental for driving SPI experiments at LCLS. These types of experiments use coherent diffraction imaging to reconstruct a 3D image of particles, including viruses (Ekeberg *et al.*, 2015 ▸; Lundholm *et al.*, 2015 ▸; Kurta *et al.*, 2017 ▸; Hosseinizadeh *et al.*, 2017 ▸), large macromolecular assemblies (Ekeberg *et al.*, 2024 ▸) or single cells (van der Schot *et al.*, 2015 ▸). The optimum photon energy for imaging low-*Z*, biological materials is within the tender X-ray range, which forms the best compromise between the scattering cross-section and the resolution of the reconstructed image. The megahertz repetition rate of the LCLS-II facility is expected to tremendously benefit the SPI technique, as the high signal-to-noise ratio (resulting from the relatively low scattering cross-section) can be improved by averaging a large number of diffraction patterns (Poudyal *et al.*, 2020 ▸).

#### 7.2.1. Data systems

With the high-energy upgrade of LCLS-II and the associated coming online of ultra-high-repetition-rate imaging detectors, the raw data throughput from crystallography experiments at LCLS will drastically increase from today’s 1–5 GB s^−1^ to north of 200 GB s^−1^, before reaching multiple TB s^−1^ in a second development phase. This puts pressure on LCLS data systems on various levels, from the timing system that triggers and builds events from multiple data streams to the data transfer and storage system which will not be able to accommodate this deluge of data without innovative compression remedies either directly on the detector pixels or upon writing to disk. A key strategic component in addressing this data deluge is the *Data Reduction Pipeline* (*DRP*), a dedicated computing infrastructure located between the instrument and the actual computing facility where long-term storage and offline processing happen. The *DRP* provides the LCLS data systems with the capacity to reduce the data volume on-the-fly using an experiment-specific compression strategy that provides 10–100× relief on downstream data volume (Thayer *et al.*, 2024 ▸).

By reducing overall throughput and storage requirements through experiment-specific veto, compression and feature extraction, *DRP* provides several benefits beyond simplifying infrastructure maintenance costs. First, it speeds up data movement to offsite computing facilities, such as NERSC or DOE’s Leadership Computing Facilities, thus faciliting colocation of data and computation for a variety of data-processing tasks, machine-learning model training or data-driven modeling projects. Second, it provides a heterogeneous playground spanning from intelligent detector pixels, to field programmable gate arrays, to GPUs and CPUs where creative algorithmic solutions can be fine-tuned to particular needs with varying degrees of timeliness or intensiveness. Finally, that playground can be used for timely extraction of actionable features sent to users or ML agents on the instrument, enabling experiment steering.

Another consequence of increased data rates is, as mentioned above, a potential explosion of parameter space exploration, in terms of both sampling density and dimensionality – more sampling density means, for example, finer sampling of the time-axis for TR experiments, while dimensionality increase means that instrument users and operators might be allowed to shift from a scarsity to an abundance mindset – instrument parameters that were carefully set ahead of time before might become random variables to be optimized constantly, to minimize drift or to limit sample damage, for example. However, to fully realize both potentials, not only do timely feature extraction and other experiment steering tools become a potentially interesting concept but they become critical for efficient decision making.

## 8. Conclusions

Over a decade and a half, the LCLS has made remarkable contributions to the field of biological sciences, offering novel ways to understand complex biological phenomena.

Through the deployment of cutting-edge instruments such as the MFX and CXI, LCLS has enabled researchers to achieve unprecedented insights into the structures and dynamics of biological molecules, particularly focusing on membrane proteins, metalloproteins and the dynamics of biomolecular processes.

Membrane proteins, which are notoriously challenging to study because of their complex environments, have been successfully analyzed, leading to a better understanding of their functions and mechanisms. Metalloproteins, with their intricate roles in various biological processes, have been elucidated in ways that avoid the damage often introduced by traditional techniques. The real-time observation of fast and ultrafast structural dynamics has opened new avenues for studying the fundamental processes that sustain life at the molecular level.

These advancements realized through the LCLS are not only limited to understanding biological structures but also extend to practical applications such as drug development and the design of new biomaterials. Robust sample delivery methods and state-of-the-art technology ensure precise and efficient experimentation, which is crucial to obtaining reliable data.

The LCLS-II-HE era will look forward to the development of new instruments MFX-HE, CXI-HE and TXI that will enable high-repetition rate experiments in the biological sciences. The concomitant increase in data scales associated with high repetition rate science will require cooperation of the leadership class high-performance computing (HPC) facilities in the United States and coordination on a national level between the LCLS and these facilities will facilitate managing and analyzing the vast amounts of data generated. Collaborative efforts in data reduction, analysis and holistic data access for meta-analysis are essential to extract the most scientific insight from LCLS data and fostering groundbreaking discoveries. The better integration of more complex computational methods, such as density functional theory and quantum mechanics/molecular mechanics approaches is anticipated to further enhance the analysis capabilities and breakthroughs from LCLS-II-HE data.

The LCLS stands as a pivotal resource in advancing both fundamental biological knowledge and applied sciences. Its continued development and the growing cooperation among scientific communities promise to drive even more significant discoveries, ensuring that LCLS and LCLS-II-HE will remain at the forefront of biological research and innovation.

## 9. Facility access

LCLS instruments are open to academia, industry, government agencies and research institutes worldwide for scientific investigations. There are typically two calls for proposals per year and an external peer-review committee evaluates proposals based on scientific merit and instrument suitability. There are also typically calls for shorter protein crystal screening beam times, which may request either the standard CXI or standard MFX endstations. Access is without charge for users who intend to publish their results. The facility is equipped to assist in all aspects of the experiment, from planning, sample preparation and delivery, to data collection and analysis. Prospective users are encouraged to contact instrument staff members to learn more about the science and capabilities of the facility, as well as opportunities for collaboration.
